# Visible wavelength spectral tuning of absorption and circular dichroism of DNA-assembled Au/Ag core–shell nanorod assemblies†

**DOI:** 10.1039/d1ma01211h

**Published:** 2022-02-21

**Authors:** Mihir Dass, Lilli Kuen, Gregor Posnjak, Sven Burger, Tim Liedl

**Affiliations:** Faculty of Physics and Center for NanoScience (CeNS), Ludwig-Maximilians-University Geschwister-Scholl-Platz 1 80539 Munich Germany tim.liedl@physik.lmu.d; Computational Nano Optics, Zuse Institute Berlin 14195 Berlin Germany

## Abstract

Plasmonic nanoparticles have unique properties which can be harnessed to manipulate light at the nanoscale. With recent advances in synthesis protocols that increase their stability, gold–silver core–shell nanoparticles have become suitable building blocks for plasmonic nanostructures to expand the range of attainable optical properties. Here we tune the plasmonic response of gold–silver core–shell nanorods over the visible spectrum by varying the thickness of the silver shell. Through the chiral arrangement of the nanorods with the help of various DNA origami designs, the spectral tunability of the plasmon resonance frequencies is transferred into circular dichroism signals covering the spectrum from 400 nm to 700 nm. Our approach could aid in the construction of better sensors as well as metamaterials with a tunable optical response in the visible region.

## Abstract 2

Plasmonic nanoparticles with Ag shells have unique properties which can be harnessed to manipulate light at the nanoscale.

## Graphical abstract

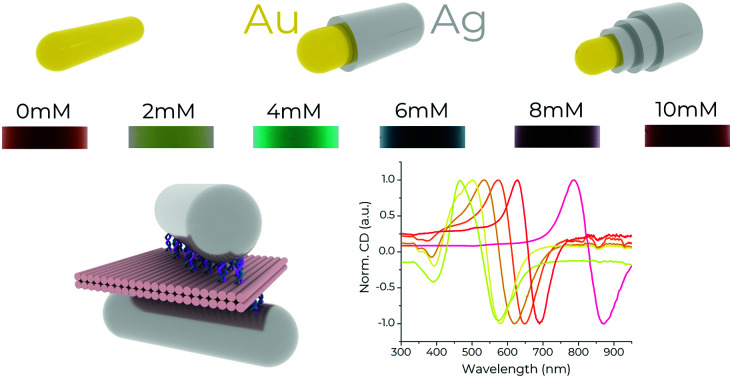

## Introduction

Circular dichroism (CD) is the differential extinction of light of different handedness by chiral materials, and as such, serves as a fingerprinting method for the characterisation of biomolecules like proteins^1,2^ and DNA.^3,4^ However, the magnitude of CD displayed by these biomolecules is low and is generally exhibited in the UV region.^5,6^ Metals such as gold interact much more strongly with light and can be useful in studying chirality.^7–9^ A wide variety of artificial chiral nanostructures have been presented using quantum dots,^10–12^ silica^13^ and metallic nanoparticles.^8,14,15^ DNA nanotechnology, due to its programmability, has emerged as an elegant tool to enable precise placement of nanoscale objects such as plasmonic nanoparticles,^16–20^ biological materials such as antibodies,^21,22^ enzymes^23^ and proteins,^24,25^ as well as polymers.^26,27^ DNA origami enables the construction of chiral structures using nanoparticles that exhibit strong CD in the visible region.^28–30^ These and other studies^31,32^ increase comprehension of the underlying near field processes responsible for the emergence of plasmonic circular dichroism. With improving design and manufacturing, chiral arrangements of gold nanoparticles have been employed for novel molecular sensing approaches^33^ and the construction of metasurfaces.^34^ However, gold nanoparticles are limited in spectral tunability. For example, spherical gold nanoparticles absorb in the 520–600 nm region^35^ whereas gold nanorods, which allow the construction of strongly chiral objects due to their anisotropic nature,^33,36–39^ usually absorb in the NIR-IR region.^40,41^ On the other hand, silver is an excellent plasmonic material exhibiting lower optical losses^42^ and a greater extinction cross-section compared to gold.^43^ Chiral assemblies constructed with silver nanorods are also predicted to have stronger chiral plasmonic interactions compared to gold.^44^ Despite these advantages, the application of silver particles in chiral plasmonics has been limited, partly due to the susceptibility to oxidation of the silver surfaces in aqueous solutions or even salty buffers required in DNA assembly.

We recently reported the synthesis of gold/silver core/shell nanorods (Au/AgNRs) functionalised with DNA in a one-pot reaction.^45^ Au/AgNRs with different silver shell thicknesses could be constructed, thus allowing spectral tuning of the nanorods. The DNA coating serves a dual purpose: it imparts additional stability to the nanorods and makes the nanorods functional to allow their assembly on a DNA origami template. Chiral assemblies constructed from the Au/AgNRs exhibit stronger g-factors compared to assemblies constructed solely from gold nanorods (AuNRs). Here we expand on that work by illustrating another plasmonic chiral enantiomer in an ‘L-shaped’ geometry and showcasing the spectral tuning of the circular dichroic signal in the visible region of the spectrum. Because of their increased stability, Au/AgNRs can serve as a viable alternative to AuNRs and expand the parameter space in future studies focused on exploring the nature of plasmonic chirality.

## Experimental methods

### DNA origami folding and purification

Staple strands (Integrated DNA Technologies, 200 μM each in water) and the scaffold strand (8064 nt long M13mp18 ssDNA) were mixed to a target concentration of 200 nM for each staple and 25 nM for the scaffold in 16 mM MgCl_2_, 10 mM Tris and 1 mM EDTA. The mixture was divided into 100 μL aliquots in PCR tubes and annealed from 65 °C to 20 °C over ∼16 hours (see ESI†). The origami structures were purified using 100 kDa MWCO Amicon filters (Millipore).

### Gold and silver/gold nanorod synthesis

The gold nanorods were synthesised as in ref. 41 The procedure was optimised for the desired size (see supplementary Note S1, ESI†). The rods were then washed in 0.1 M CTAB (Roth) and 0.01 M CTAB and stored in 0.01 M CTAB at 15 OD (optical density) before use. For the silver coating, the AuNRs were added to 0.1 M CTAB under stirring (500 rpm) and allowed to mix for 10 s. Then, thiolated ssDNA strands (Biomers, 100 μM, aq.) were added to the rod mixture. AgNO_3_ solution was added next, after which the stirring speed was increased (1500 rpm). l-Ascorbic acid (0.2 M) and NaOH (0.2 M) were added rapidly in quick succession. A colour change after 5–10 s indicates the successful synthesis of a silver coating. The reaction was allowed to proceed for 10 min. Both the AuNRs and Au/AgNRs were washed to remove excess reactants from their respective growth solutions (see supplementary note S1, ESI†) and then redispersed in 0.1% sodium dodecyl sulfate (SDS) before further use.

### DNA-nanorod hybridisation

A second DNA-functionalisation step increases the stability and binding of the nanorods. Au/AgNRs were mixed with an adequate volume of thiolated ssDNA (Biomers, 100 μM, aq.) followed by freezing @ −80 °C for 30 minutes. They were then thawed and purified from excess ssDNA using agarose gel electrophoresis. The DNA origami and functionalised nanorods were mixed to a final concentration of 1–2 nM and 20–40 nM, respectively, *i.e.* the rods were added in ∼20× excess. The mixture was then annealed from 45 °C to 20° at 10 min °C^−1^, and this ramp was repeated four times.^36^ The assemblies were then separated from excess unbound rods and aggregates using agarose gel electrophoresis.

### Characterisation

#### Agarose gel electrophoresis

The nanorods and origami–rod hybrids were analysed and purified using a 1% agarose gel. The gels were run in a buffer containing 40 mM Tris, 20 mM acetic acid, 1 mM EDTA and 11 mM MgCl_2_ at 100 V for 2 hours. 1× SybrSafe (Thermo Fisher) was included in gels for DNA origami analysis.

### UV-Vis spectroscopy

The nanorods and DNA samples were analysed using a Nanodrop-1000 spectrometer. A 1.5 μL droplet of the sample and a path length of 1 mm was used to determine the optical density (OD) values.

### TEM imaging

5 μL of a sample was incubated for 30 s–5 min, depending on concentration, on glow discharged TEM grids (formvar/carbon, 300 mesh Cu; Ted Pella) at room temperature. After incubation on the grids, the sample was wicked off by bringing the grid into contact with a filter paper strip. Nanorod samples were imaged as-is. Samples containing DNA origami went through an additional staining step with a 2% uranyl formate aqueous solution containing 25 mM sodium hydroxide. After incubating and wicking the sample off, a 5 μL drop of uranyl formate staining solution was applied to the grid, immediately wicked off, followed by applying another 5 μL drop of uranyl formate staining solution. This drop was allowed to incubate on the grid for 10 seconds and then wicked off. The grid was allowed to dry for 5 minutes before imaging. Imaging was performed with a JEM1011 transmission electron microscope (JEOL) operated at 80 kV.

### CD spectroscopy

All CD measurements were performed in a Chirascan plus CD spectrometer (Applied Photophysics) with 10 mm path length quartz cuvettes (Hellma). The cuvettes were cleaned with fresh aqua regia, washed thoroughly with MilliQ water and dried with compressed N_2_ before each measurement. The baseline measurements were performed with 40 mM Tris, 20 mM acetic acid, 1 mM EDTA buffer with 11 mM MgCl_2_. The scanning step size was set to 1.0 nm with an acquisition time of 1.0 s for each step.

### Simulation

The total extinction and CD spectra were computed numerically, solving the linear, time-harmonic Maxwell's equations. A single arrangement was included in the computational domain for each setup (single nanorod arrangement, NR–NR arrangement in ‘X-shape’, and NR–NR arrangement in ‘L-shape’). Tabulated data was used for modelling the permittivities of gold and silver.^46,47^ For the background solution, a constant permittivity of *ε*_r_ = 1.8 was assumed. In the numerical experiment, the arrangement was illuminated by pairs of left- and right-handed circularly polarised plane waves incident along the coordinate directions. The total extinction and CD spectra were derived from the near-field solution (see supplementary note S2, ESI†).

## Results and discussion

**Fig. 1:**
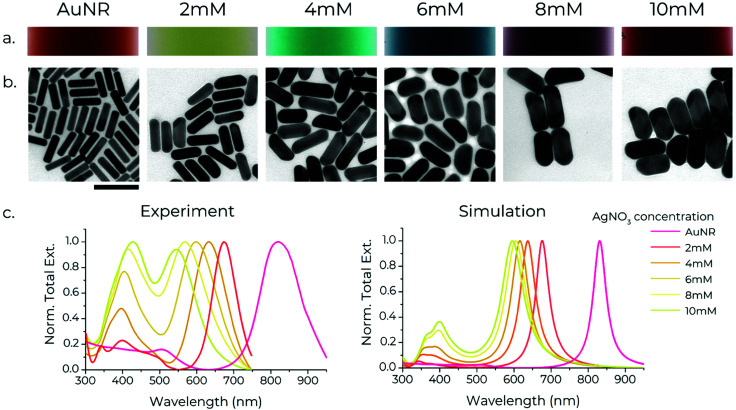
Synthesis of Au/AgNRs with varying shell thickness. (a) Variation of colour of Au/AgNR suspensions grown with different concentrations of Ag^+^ ions. (b) Transmission electron micrographs of Au/AgNRs with Ag shells of different thicknesses. The concentration of Ag^+^ in the growth solution is shown at the top. All images are to scale. Scale bar: 100 nm. (c) Experimental and theoretical extinction spectra of the AuNRs and Au/AgNRs with varying shell thickness.

Gold nanorods (AuNRs, ∼65 × 15 nm) were synthesised using a modified protocol (see supplementary note S1, ESI†) from González-Rubio *et al*.^41^ These AuNRs serve as seeds for the growth of silver shells around them using the protocol outlined by Nguyen *et al.*^45^ Specifically, AgNO_3_ (aq.) and thiolated-DNA are added to the dispersion of AuNRs. Then, in the presence of l-ascorbic acid, AgNO_3_ is reduced to metallic silver preferentially on the AuNRs, resulting in the formation of silver-coated AuNRs (Au/AgNRs). The presence of thiolated-DNA in the growth solution leads to simultaneous functionalisation of the rods. Importantly, wide and small angle X-ray scattering data indicate that the DNA conjugation occurs only on the surface, not within the shell.^45^ The shell growth proceeds in an anisotropic fashion, with the shell usually being thicker on the sides compared to the tips, which could arise from different crystal facets being exposed at the sides and tips of a nanorod.^48^ The shell growth results in a visible change of the colour of the rod suspensions, with the color varying as a function of the shell thickness (Fig. 1a). The nanorods were characterised using transmission electron microscopy and UV-Vis spectroscopy.

### Structural characterisation

TEM micrographs confirm good monodispersity of the rods before and after shell growth, with the size deviation increasing with increasing shell thickness (ESI,† Fig. S9). The AuNR ‘core’ is visible in the centre of the Au/AgNRs due to gold and silver's different electronic densities (Fig. 1b). A noteworthy aspect of the growth is that in some Au/AgNRs the shell growth is asymmetric, with the shell being thicker on one side of the rod. This is in line with previous studies which show that this asymmetry arises from preferential deposition of Ag on certain crystal facets on the Au surface, which is believed to arise from selective adsorption of CTAB along certain crystal planes of Ag.^49^ As the shells grown by this method are thicker on the sides compared to the tips, increasing the shell thickness leads to a decrease in the aspect ratio of the rods. Shells between ∼5 nm to ∼14 nm thickness (measured perpendicular to the long axis) were grown, with their aspect ratio changing from 4, in the case of bare AuNRs, to ∼2 for Au/AgNRs in the case of 14 nm shells grown with 10 mM AgNO_3_ (Fig. 1b). Growing thicker shells resulted in rods with an aspect ratio ∼1, thus making the identification of the long axis unreliable (ESI,† Fig. S14a and b).

### Optical characterisation

The absorption spectra of gold nanorods exhibit two peaks, for the transverse and longitudinal modes of localised surface plasmon resonances (LSPR) perpendicular to and along the long axis of the rods, respectively (Fig. 1c).^50^ The transverse mode usually occurs around ∼500 nm, which also corresponds to the LSPR of spherical particles.^50^ The longitudinal mode peak, at a constant thickness, shifts depending on the rods’ length (and thus the aspect ratio), with longer lengths redshifting the peak. Growth of Ag shells leads to a blueshift in the longitudinal LSPR of the rods, with thicker shells resulting in a stronger blueshift. Since the shell thickness depends on the concentration of Ag^+^ ions present during shell growth, the longitudinal LSPR can be tuned over a wide range of wavelengths.^45^ Here, we show stepwise manipulation of the longitudinal LSPR from 820 nm, *i.e.* the NIR spectral region (for bare AuNRs) down to 540 nm, *i.e.* the green spectral region (for Au/AgNRs synthesised using 10 mM AgNO_3_, Fig. 1c). We are able to create particles with thicker shells that push the longitudinal LSPR to 480 nm *i.e.* the blue spectral region. However, the shape of particles is no longer consistently anisotropic (ESI,† Fig. S14c). Numerical simulations satisfactorily replicate the experimental spectra, with increasing shell thickness blueshifting the LSPR. The LSPR peaks in the numerically calculated data are much narrower than experimental spectra because they exclude the size polydispersity of bulk nanorod samples. The agreement between the simulations and experimental spectra is remarkable for the AuNRs and Au/AgNRs with thin Ag shells. The deviation increases for Au/AgNRs with thicker shells, consistent with an observed increase in their shape inhomogeneity (see also supplementary note S3, ESI†). The large spectral gap between the LSPR of the AuNRs and the Au/AgNRs with the thinnest Ag shell (grown using 2 mM AgNO_3_) is worth noting, which is also reproduced by our simulations. Generally, we believe this effect arises as a combination of the silver shell as well as a decrease in the aspect ratio of the rods, which results in a blue-shift of the LSPR^40^ (ESI,† Fig. S10). Unfortunately, we were not able to obtain stable samples with thinner shells.

The Ag shell also results in a more complex transversal mode appearing at shorter wavelengths, with a strong peak at ∼400 nm and a secondary feature at shorter wavelengths ∼350 nm. Both of these redshift with increasing shell thickness. Simulations revealed that the latter mode arises due to the transversal excitation of the Au/AgNRs, leading to the formation of charged dipoles between the Au–Ag interface and the outer surface of the Au/AgNRs.^45,51^ This secondary peak increases in intensity with increasing Ag shell thickness and becomes more intense than the longitudinal mode in Au/AgNRs with very thick shells. The appearance of this secondary mode agrees very well with the simulations (Fig. 1c), with the simulations reproducing the spectra – including the increase in intensity and redshift of the new transverse peak with increasing shell thickness.

### Chiral metamolecule fabrication

**Fig. 2:**
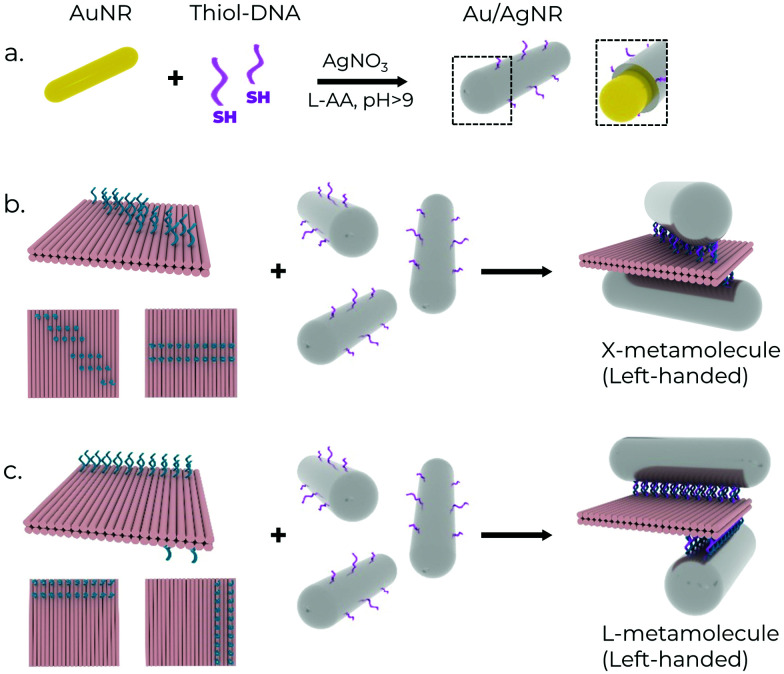
Schematic showing the fabrication of the chiral metamolecules. (a) One-pot growth and functionalisation of the Ag shells on AuNRs. (b) and (c) Assembly of ‘X’ and ‘L’-shaped chiral structures, respectively, constructed by varying the anchor configuration (blue strands) on the 2-layer sheet DNA Origami (left). The ‘upper’ (left) and ‘lower’ (right) faces of the sheet are shown below the respective sheet structures.

AuNRs and Au/AgNRs were functionalised with DNA for attachment to DNA origami templates using DNA anchors (Fig. 2a). We utilised the freeze-and-thaw method, which is an effective and facile method for functionalising various plasmonic particles.^52–54^ While the DNA strands attached to the Au/AgNRs during the shell growth already provide long-term stability, an additional freeze-and-thaw functionalisation step is performed to ensure a dense coverage of the nanorod surface with DNA strands. Different volumes of thiolated DNA were added to AuNRs (at OD 15) suspended in 0.1% SDS for the functionalisation optimisation. The mixture was vortexed together and frozen at −80 °C for 30 min. We aim to cover the nanorods with a dense blanket of DNA oligos to maximise their long-term stability in the salt conditions conventionally used for DNA origami buffers (typically ∼12 mM MgCl_2_), and maximise their hybridisation probability with DNA origami.^55^ The successful functionalisation of the rods is verified by agarose gel electrophoresis, which also serves as a purification method for separating the functionalised rods from excess oligonucleotides. Successfully functionalised rods travel as a single band in the gel and can be separated from aggregates and unfunctionalised rods (ESI,† Fig. S1a).

**Fig. 3:**
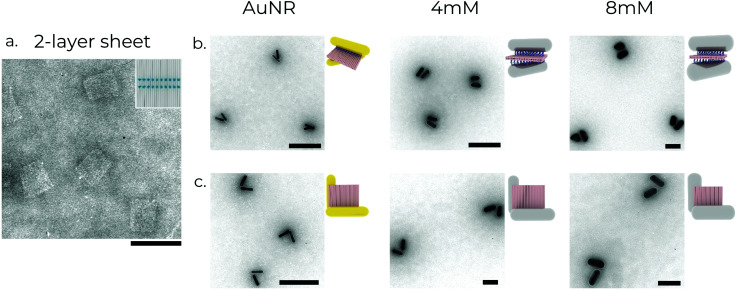
Structural characterisation of chiral metamolecules. (a) Transmission electron micrograph of the 2-layer sheet DNA origami structures. (b) and (c) X- and L-shaped left-handed chiral structures assembled using three varieties of nanorods, respectively. The concentration of AgNO_3_ used to synthesise the nanorods used for the structures is shown above the images. All scale bars: 100 nm.

While these bimetallic Au/AgNRs possess novel optical properties, utilising the high degree of addressability of DNA origami to create predefined assemblies offers the opportunity to study more complex physical phenomena, *e.g.* plasmonic chirality. A square 2-layer DNA origami sheet (side length ∼55 nm, thickness ∼5 nm) was used as a template to construct the chiral metamolecules. 16–20 anchors with 8 nt long poly-A sequences at the 3′ end were extended from opposite faces to act as linkers for the nanorods. The layout of the anchors determines the final chirality of the resultant assembly. Two chiral geometries were utilised in this manuscript (Fig. 2b and c). In the first, the anchor positions on the ‘upper’ face were rotated by ∼50° relative to the ‘lower’ face to obtain a left-handed ‘X’-shaped rod assembly, resulting in strongly asymmetric optical performance.^45^ In the second, the anchor positions on the two faces were rotated 90° around one corner to obtain a left-handed ‘L’-shaped assembly. It is worth mentioning that while the ‘X’-configuration is chiral only if the angle between the two nanorods ≠90°, the ‘L’-configuration has broken inversion symmetry, so it is chiral even when the nanorods are perpendicular to each other. For the hybridisation reaction, amicon-purified DNA origami was slowly pipetted into a tube containing purified functionalised rods under vortexing.^56^ The aim here is to introduce the DNA origami to a ‘reactant’ (nanorods) present in excess to maximise the chances of dimer formation and minimise the formation of spurious aggregates by saturating the binding sites on the DNA origami. The resulting mixture was then annealed using a modified protocol (see Supplementary Note S1, ESI†) to maximise binding yield and prevent partially bound rods.^36^ The metamolecule dimers were separated from unbound excess nanorods and aggregates using agarose gel electrophoresis due to their different migration speeds in the gel matrix (ESI,† Fig. S1b). The relevant gel bands were excised and squeezed between a glass slide and parafilm to extract the liquid sample, on which further measurements were performed. TEM imaging of the gel-purified sample showed the successful assembly of the desired metamolecule configurations with a high degree of fidelity (Fig. 3).

### CD spectroscopy

**Fig. 4:**
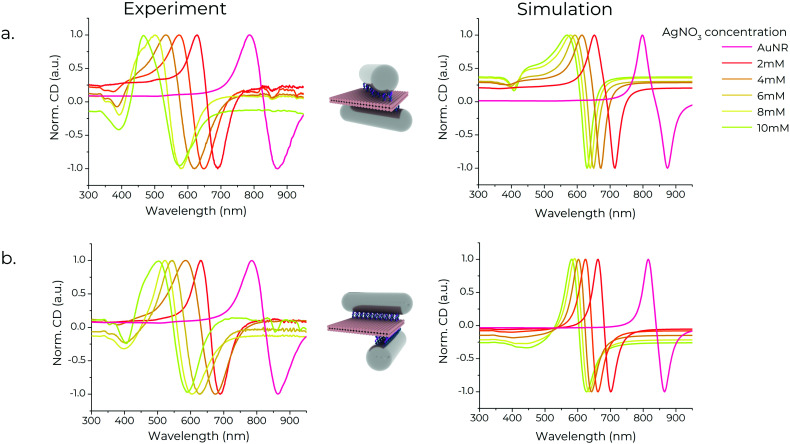
Experimental (left) and theoretically calculated (right) CD spectra of the (a) X-shaped and (b) L-shaped chiral metamolecules constructed using Au/AgNRs with Ag shells of varying thicknesses. The legend shows the concentration of AgNO_3_ used to synthesise the nanorods in the metamolecules.

We performed CD spectroscopy of chiral metamolecules purified with agarose gel electrophoresis. The absorption during CD measurements was set to ∼0.6–0.7. The spectra of the chiral metamolecules, shown in Fig. 4, indicate a strong chiral signal, noteworthy being the signature peak-to-dip bisignate profile of the spectra.^36–39,57^ This profile arises from plasmon-mediated near-field interactions of the individual NRs in each assembly when excited by circularly polarised light. A plasmonic dipolar moment is induced in the rods when excited by light. This results in the rise of two distinct modes with opposite chirality – namely a high-energy and a low-energy mode – centred around the longitudinal mode of the nanorods.^58^ The high-energy mode is blue-shifted compared to the resonance of the individual nanorods, while the low-energy mode is red-shifted (ESI,† Fig. S15). The CD spectra of chiral metamolecules assembled with Au/AgNRs blueshifts with increasing silver shell thickness, in line with the longitudinal resonances of the corresponding Au/AgNRs. This trend of the CD response is in accord with the simulations (Fig. 4). In chiral metamolecules constructed with Au/AgNRs, additional signatures appear between 300–400 nm, also seen in the simulations. A possible explanation for this is the coupling of the transverse modes seen in Au/AgNRs, as this CD signature appears in the same spectral region and increases with increasing Ag shell thickness similar to the individual Au/AgNRs. Another reason could be the coupling of multipolar plasmonic modes observed in anisotropic silver nanostructures, although they occur at longer wavelengths compared to our structures.^59–61^ The lineshape of the experimental spectra is also qualitatively in accord with simulations, noteworthy being the various dips around 400 nm that differ between the X- and L-shaped dimers. This feature is broader and less pronounced in simulations and experiments for the L-shaped dimer. At the same time, we observed a positive signal over the entire blue part of the spectrum for the X-shaped structures. The bespoke dip only appears for thicker silver shells, again nicely reproduced in the simulations. Overall, the differences are more pronounced in theory than in experiments, which we ascribe to size polydispersity of the rods and shape polydispersity in the dimers themselves. For example, while designed to have a 90° angle between the rods, the L-shaped dimers often exhibit lower angles (ESI,† Fig. S4).

## Conclusions

We have developed a class of core–shell nanorods composed of Au and Ag, which allows for fine control over the spectral tuning of the optical response on the visible spectrum. Moreover, chiral metamolecules constructed with these Au/AgNRs extend the spectral control over the CD signal. We present data where we tune the longitudinal LSPR of AuNRs – initially 819 nm – from 673 nm to 544 nm. The extent of this control depends on the aspect ratio of the AuNR core. Using AuNRs with a longitudinal LSPR farther in the IR would allow the manipulation of the longitudinal mode in the IR-NIR region by employing Ag shell growth. We also see the appearance of new spectral features in the 300–400 nm wavelength region. We provide theoretical simulations that show remarkable agreement with the experimental results, both for the extinction of Au/AgNRs and the CD from chiral metamolecules constructed with the Au/AgNRs. Au/AgNRs have gained popularity in recent times as a novel plasmonic material.^51^ Chiral metamolecules constructed using Au/AgNRs can result in strong chiral signals^45^ and could be used for sensitive detection.^33^ In addition, the freedom to tune the optical response can be used to synthesize metamaterials with stronger optical activity in the visible region. The tunability can also be used to modulate the optical response to enhance LSPR-dependant coupling to other moieties, *e.g.* dyes,^62^ and also open the doors for the realisation of other applications, *e.g.* chiral reactions.^51,63^ Additionally, our protocol should be extendable to more exotic morphologies of gold particles as well, *e.g.* nanostars,^64^ chiral particles^65^ or nanocubes.^66^

## Author contributions

MD and TL conceived the experiments. MD designed the DNA origami structures and performed the experiments. LK performed the theoretical simulations. MD and GP analysed the experimental data and wrote the manuscript. All authors discussed the results and commented on the manuscript.

## Conflicts of interest

There are no conflicts to declare.

## Supplementary Material

MA-003-D1MA01211H-s001
