# Supplementary material for: Visible wavelength spectral tuning of absorption and circular dichroism of DNA-assembled Au/Ag core–shell nanorod assemblies
Source: Mater Adv. 2022 Feb 21;3(8):3438–45. doi: 10.1039/d1ma01211h (PMC9017759; doi:10.1039/d1ma01211h)
Supplement: MA-003-D1MA01211H-s001 [file MA-003-D1MA01211H-s001.pdf]

Please do not adjust margins

Electronic Supplementary Material

Visible wavelength spectral tuning of absorption and circular dichroism of DNA-assembled Au/Ag core-shell nanorod assemblies

Mihir Dass, Lilli Kuen, Gregor Posnjak, Sven Burger and Tim Liedl *

Supplementary Note S1 - Experimental Section

Synthesis of AuNRs

The synthesis protocol was adapted from[1]. The protocol was repeated with different ratios of the reagents until AuNRs of the desired size were synthesised. The final synthesis recipe is below :

Reagent	Concentration	Volume
CTAB - n-decanol		90mL
HauCl4	50mM	900uL
AgNO3	10mM	1800uL
AA	0.2M (weight x 56.8)	720uL
HCl	1M	3600uL
Seed	OD 0.6 at 400nm	2880uL

Washing AuNRs

AuNRs were washed 2x in 0.1M CTAB, and then were redispersed in 0.01M CTAB for further use. For AuNRs to be used for functionalisation with thiol-DNA, the rods were washed and redispersed in 0.1% SDS.

Synthesis of Au/AgNRs nanorods

Reagent	Concentration	Volume
CTAB	0.1M	2250 uL
AuNRs	15 O.D. in 0.01M CTAB	480uL
Thiol-DNA	T8:T19 (9:1)<br>100uM in water	480uL
$AgNO_3$	2-12mM	400uL
L-Ascorbic acid	0.2M	64uL
NaOH	0.2M	125uL

$AgNO_3$ conc. added	Actual $AgNO_3$ conc. in growth sol.
2mM	210.5 µM
4mM	421 µM
6mM	631.7 µM
8mM	842 µM
10mM	1053 µM

Washing AuAgNRs

1. The synthesised rod dispersion was aliquoted into 300uL batches.
2. The rods were centrifuged and then redispersed in 0.1M CTAB. Centrifugation parameters were 4500rcf, 5min for rods synthesised with 2-6mM $AgNO_3$, and 3000rcf, 3min for rods synthesised with 8-12 mM $AgNO_3$.
3. The rods were centrifuged and then redispersed in 0.01M CTAB.
4. The rods were centrifuged and then redispersed in 0.1% SDS.

Note : Aggregation of the AuAgNRs most commonly occurred during the washing steps. If the centrifugation speed was too high, the AuAgNR pellet had a metallic lustre and was difficult to redisperse. For washing, we advise starting from lower centrifugal speeds and gradually increasing them until a clear supernatant is obtained.

Functionalisation of nanorods

Functionalisation was found to work best when the nanorods were suspended in 0.1% SDS.

Please do not adjust margins

Please do not adjust margins

AuNRs

32uL of thiol-T19 and 288uL of thiol-T8 were mixed together and added to 480uL of AuNRs dispersed in 0.1% SDS. The mixture was vortexed for 5s, and kept at -80°C for 30min. The 800uL mixture was then thawed and centrifuged to concentrate it down to ~100uL. Agarose gel electrophoresis was used to separate the functionalised AuNRs from excess thiol-DNA. A 1% gel was cast and the run parameters were 100V, 250mA, 1hour. The relevant gel bands were excised and squeezed between a glass slide and parafilm to extract the liquid sample.

AuAgNRs

In a typical experiment, after washing, the rods were centrifuged and resuspended in 0.1% SDS to a final volume of 480uL. 32uL of thiol-T19 and 288uL of thiol-T8 were mixed together and added to the rod dispersion. The mixture was vortexed for 5s, and kept at -80°C for 30min. The 800uL mixture was then thawed and centrifuged to concentrate it down to ~100uL. Agarose gel electrophoresis was used to separate the functionalised AuNRs from excess thiol-DNA. A 1% gel was casted and the run parameters were 100V, 250mA, 1 hour in 'Gel-buffer' (40mM Tris, 20mM Acetic acid, 1mM EDTA and 11mM Mg^{2+}). The relevant gel bands were excised and squeezed between a glass slide and parafilm to extract the liquid sample.

DNA Origami

Staple strands and the scaffold strand (modified 8064 nt long M13mp18 ssDNA) were mixed together to a target concentration of 200nM and 25nM respectively in 16mm $MgCl_2$, 10mM Tris and 1mM EDTA. The mixture was divided into 100uL aliquots in PCR tubes and annealed from 65°C to 20°C over ~ 16 hours (detailed program below).

**Temperature (^{o}C)**	**Time**
65	15 min
64-60	5 min each
59-40	45 min each
39-36	30 min each
35-20	5 min each

The origami structures were purified using 100kDa MWCO Amicon filters.

1. Wet the filter by adding 450 µL water.
2. Spin filter at 4000rcf for 3 min. Discard the filtrate.
3. Add 200µL of the folded origami and 250 µL 'TE-12.5' buffer (10mM Tris, 1mM EDTA and 12.5mM Mg^{2+}). Spin at 6000 rcf for 6 min.
4. Discard the filtrate. Add 420 µL *TE-12.5* buffer and spin at 6000 rcf for 6 min.
5. Repeat step (4) three more times.
6. Invert the filter into a clean tube and spin at 6000 rcf for 2 min to collect the purified origami.

All experiments in this manuscript were performed using a single batch of 800uL origami (for both the X-shape and L-shape structures) which was folded and purified using this method above. After amicon purification, the origami were diluted to a concentration of 30nM.

Nanorod-origami hybridisation

50uL aliquots were made for each hybridisation experiment. Agarose gel purified nanorods were centrifuged down and resuspended in *Gel-buffer* to a final volume of ~ 48.5uL. 1.5uL of the origami was added to the nanorods while vortexing the sample.[(2)] The samples were then cycled from 45°C to 20°C at 10min/^{o}C a total of 4x.[(3)]

The samples were then run in a 1.2% agarose gel and the run parameters were 100V, 250mA, 2 hours in *gel buffer*. The relevant gel bands were excised and squeezed between a glass slide and parafilm to extract the liquid sample.

This journal is © The Royal Society of Chemistry 20xx

Please do not adjust margins

Please do not adjust margins

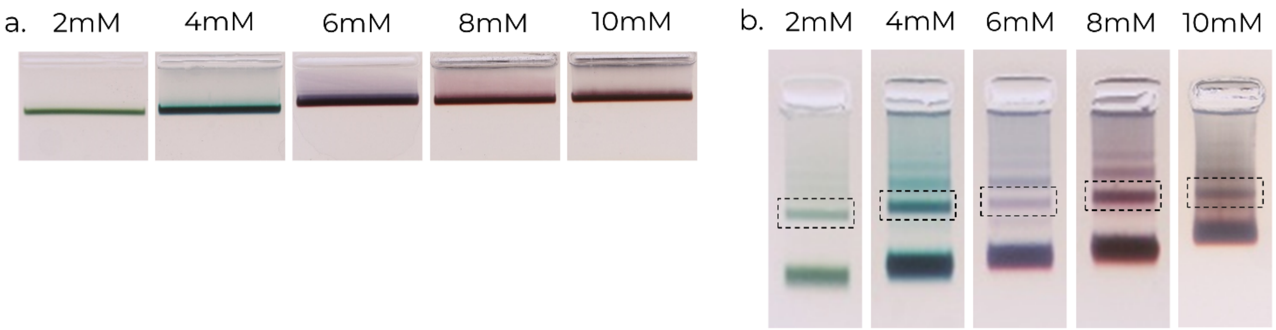

Figure S1. Agarose gel electrophoresis of a) AuAgNRs of varying Ag shell thickness and b) the X-shape chiral metamolecules constructed from the AuAgNRs. The Ag^+ concentration used for synthesising the Ag shells is mentioned above the respective lane.

TEM sample preparation

TEM grids were exposed to Argon plasma for 30s before sample deposition. The deposition duration for DNA origami (~ 5nM) was 30s, and for the NR-origami structures was 15min. The grid was then dipped on a 5uL droplet of 2% Uranyl formate (UFo) solution (containing 25mM NaOH) which was then wicked off immediately. The grid was then dipped in another UFo droplet, incubated for 10s and then wicked off. It was allowed to dry before imaging.

TEM images – Nanorods

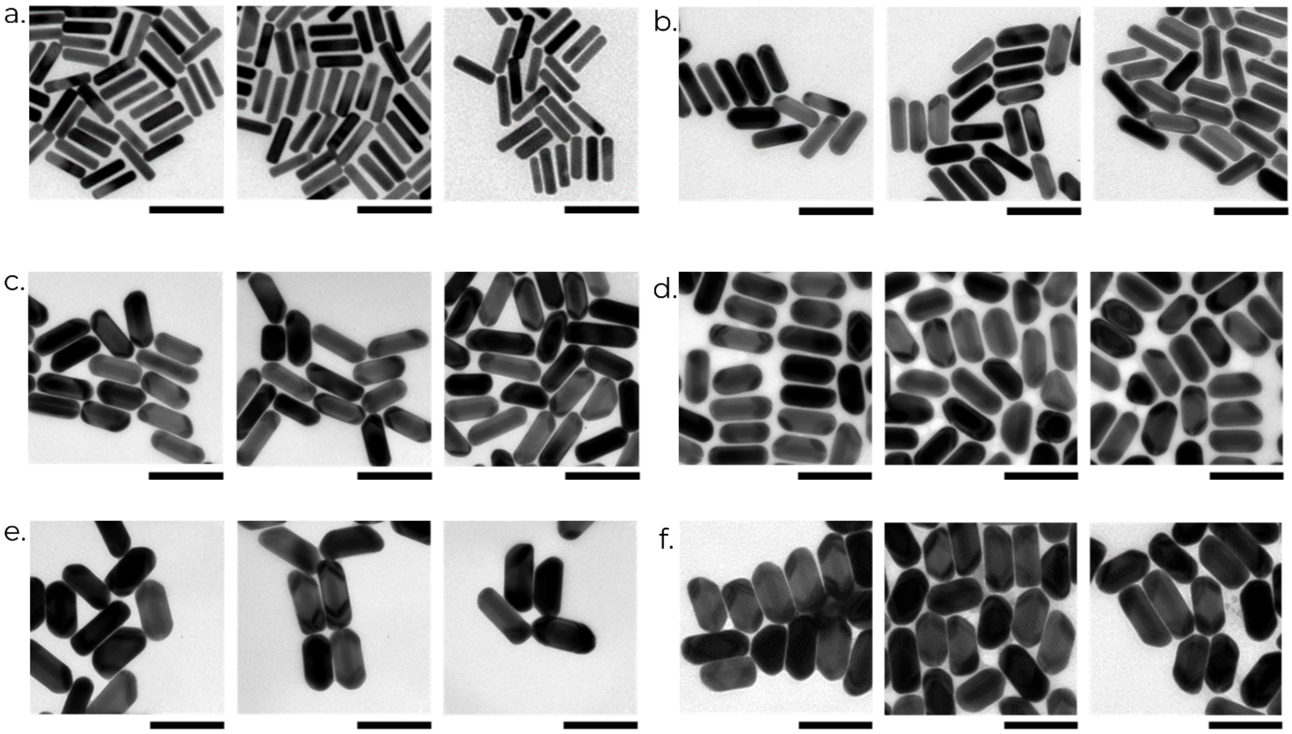

Figure S2. TEM images of nanorods with varying shell thicknesses. a) AuNRs. Au/AgNRs synthesized using b) 2mM, c) 4mM, d) 6mM, e) 8mM, f) 10mM of Ag^+ added to the growth solution. All scale bars are 100nm.

This journal is © The Royal Society of Chemistry 20xx

Please do not adjust margins

Please do not adjust margins

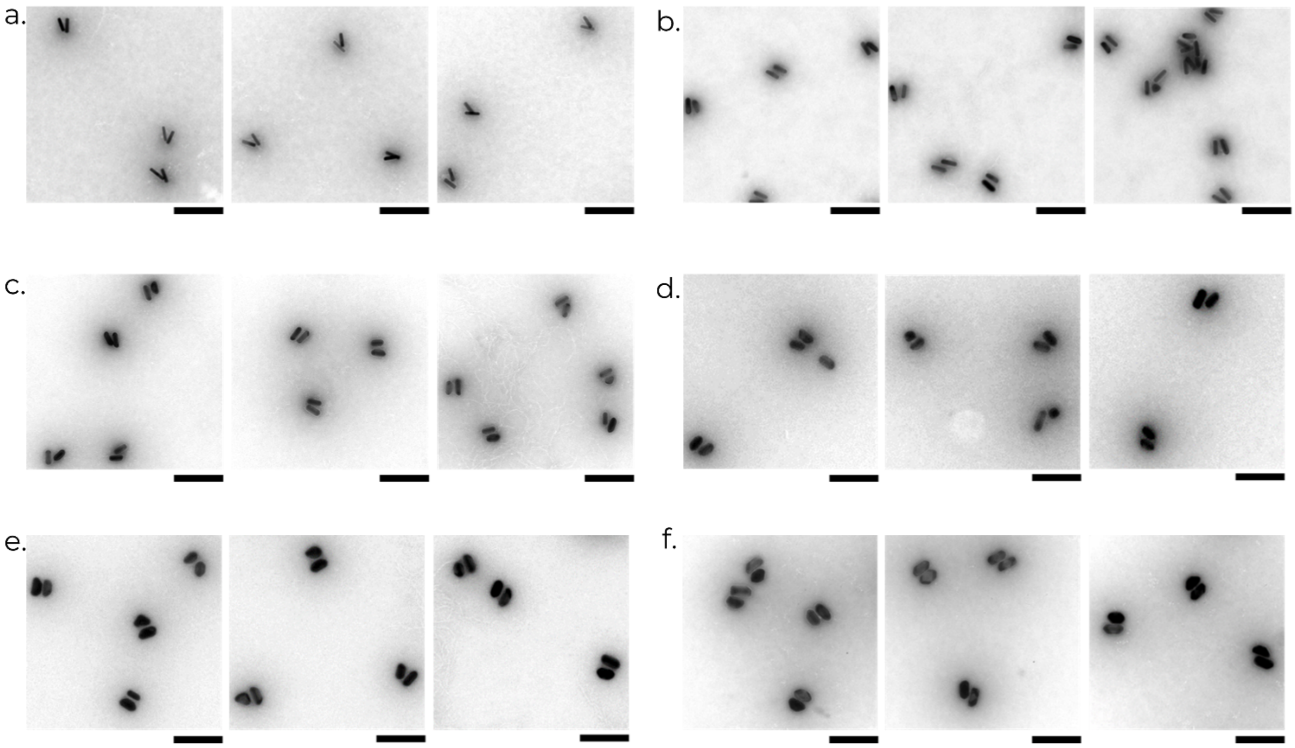

Figure S3. TEM images of X-shape chiral metamolecules assembled with a) AuNRs; Au/AgNRs synthesized using b) 2mM, c) 4mM, d) 6mM, e) 8mM, f) 10mM of Ag^+ added to the growth solution. All scale bars are 200nm.

TEM images – Chiral metamolecules (X-shape)

 This journal is © The Royal Society of Chemistry 20xx

Please do not adjust margins

Please do not adjust margins

TEM images – Chiral metamolecules (L-shape)

Supplementary note S2 – Numerical Section

Numerical Simulations

Numerical simulations were performed using the finite-element method (FEM), implemented in the solver JCMsuite.(4) Within all simulations, a polynomial degree of the FEM ansatz function p=2 was used. The discretisation was realised by a tetrahedral mesh with edge sizes smaller than 16 nm for the background, 4nm for the Ag shell, and 3.5 nm for the Au NPs. Transparent boundary conditions were realised using perfectly matched layers (PML).

Each side of the computational domain was illuminated by one left and one right-handed circularly polarised plane wave (LCP and RCP). The absorbed electromagnetic field energy and the electromagnetic field energy scattered outwards were recorded for each source. The total extinction was given by the sum of absorption and scattering for LCP and RCP, and all six directions of incidence. All graphs of the total extinction were normalised to their maximum value. The CD spectrum was given as the difference of extinction of LCP and of RCP. The graphs of the CD were normalised to their maximum to dip value, corresponding to the curves of the experimental data.

The simulation results, shown in Fig. 1 and Fig. 4 were computed using a scripting language (Matlab), to auto-generate the input files and to distribute the FEM computations to various threads on a workstation for parallel computation of the wavelength and parameter scans.

Geometry

The basic geometry for a single coated rods is depicted in Fig. S5a. It consists of an inner rod made of gold with diameter $d_i = 15$ nm and height of $h_i = 61$ nm and an outer shell with diameter d_o and height h_o, that represents the silver coating. The thickness of the silver coating is given as t_d at the side of the rods and t_h at the top, in the case of homogeneous coating the thickness is given as $t_{sh} = t_d = t_h$. The cap rounding radii of rod and shell are $d_i/2$, resp. $d_o/2$. Three different arrangements were analyzed, a single NR and NR-NR arrangements in X- and L-shape. The thickness of the silver coating was varied within the different cases for each arrangement. The sizes for the outer rod are given in Table S1 for the graphs of the numerical simulation in. 1 and Fig. 4, and in Table S2 for the graphs in Fig. S6 and Fig. S7 in this supplementary section. The NR-NR arrangement in X-shape, see Fig. S5b consists of two rods, with the rear one rotated 45° counterclockwise. The NR-NR arrangement in L- shape, shown in Fig. S5c consists of two rods, with the lower one rotated 90° counterclockwise. The offset between the rods is 11 nm from surface to surface, for both arrangements, X- and L-shape.

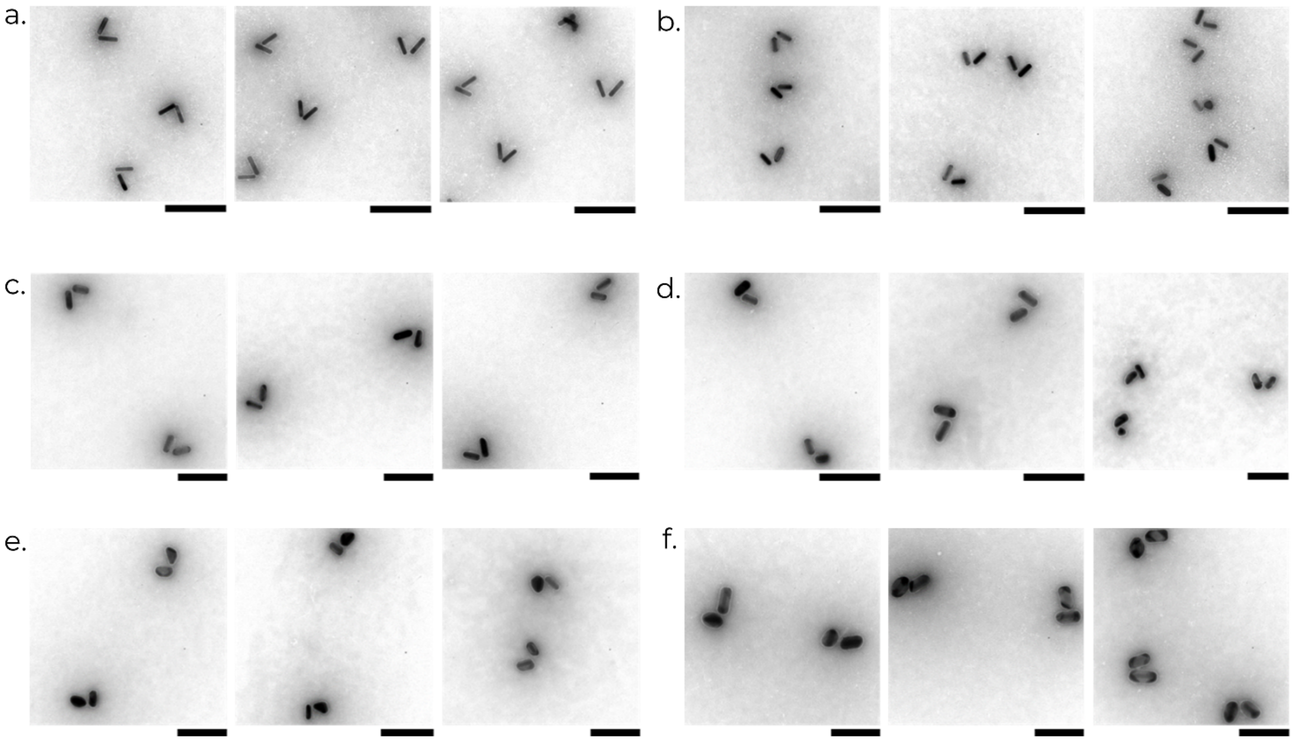

Figure S4. TEM images of L-shape chiral metamolecules assembled with a) AuNRs; Au/AgNRs synthesized using b) 2mM, c) 4mM, d) 6mM, e) 8mM, f) 10mM of Ag^+ added to the growth solution. All scale bars are 200nm.

This journal is © The Royal Society of Chemistry 20xx

Please do not adjust margins

Table S1. Measured sizes of the outer rod for all three arrangements, corresponding to the simulations for Fig. 1 and Fig. 4 with the concentration of $AgNO_3$ in [mM] in the growth solution, thickness of the silver coating at the side of the rods t_d and the tips t_h, the diameter d_o and height h_o of the outer rod, representing the silver shell.

Case	1	2	3	4	5
$AgNO_3$ [mM]	2	4	6	8	10
t_d [nm]	4.9	7.5	9.5	12.5	13.8
t_h [nm]	4.5	6.5	8	11.5	12.25
d_o [nm]	24.8	30	34	40	52,6
h_o [nm]	70	74	77	84	85.5

Table S2. Homogenous silver coating with $t_{sh} = t_d = t_h$ starting from 5 nm in 2 nm steps up to 33 nm, corresponding to the simulations of Fig. S6 and Fig. S7 and the resulting diameter $d_o$and height h_o of the outer rod.

Case	1	2	3	4	5	6	7	8	9	10
$t_{sh} = t_d = t_h$ [nm]	5	7	9	11	13	15	17	19	21	23
d_o [nm]	25	29	33	37	41	45	49	53	57	61
h_o [nm]	71	75	79	83	87	91	95	99	103	107

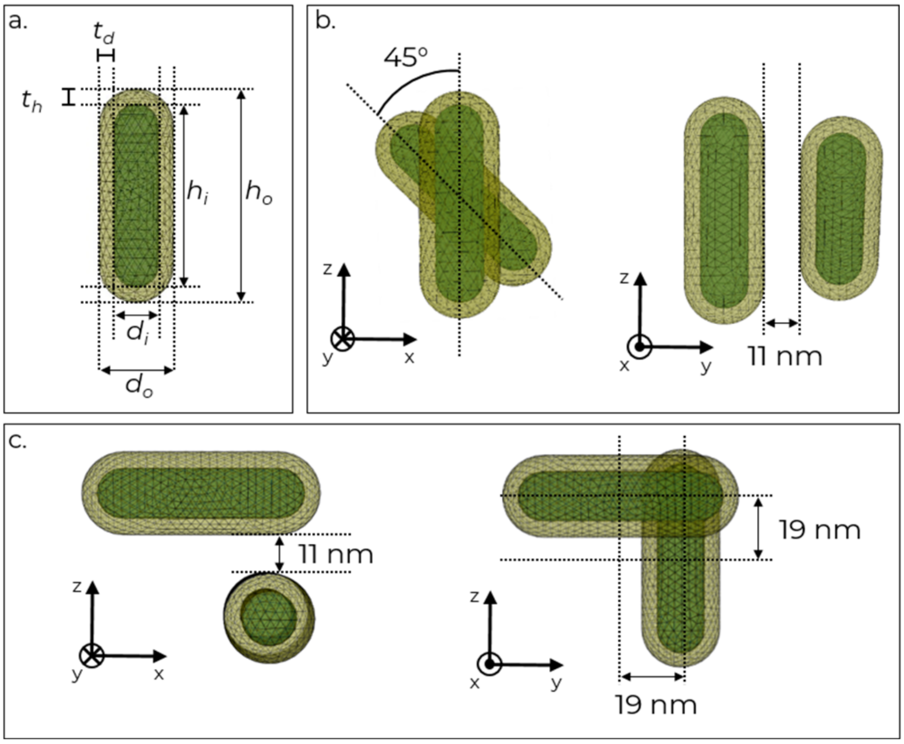

Figure S5. a) Single gold rod with diameter $d_i = 15$ nm and height $h_i = 61$ nm and silver shell with thickness t_d at the side of the rod and t_h at the top, represented by an outer rod diameter d_o and height h_o. The cap rounding radii of rod and shell are $d_i/2$, resp. $d_o/2$. b) NR-NR arrangement in X-shape with 45° angle between the NRs and 11 nm distance between the surfaces of the NRs. c) NR-NR arrangement in L-shape with 90° angle between the NRs and 11nm distance from surface to surface of the NRs.

 This journal is © The Royal Society of Chemistry 20xx

Parameter studies with homogeneous coating

We study the influence of the silver coating, performing a parameter study for various silver shell thicknesses for all three arrangements. Therefore, we assume a homogenous silver coating $t_{sh} = t_d = t_h$ and vary the thickness of the shell t_{sh} from 5nm to 23nm in 2nm steps, sizes given in Table S2. The normalised total extinction in dependence on the wavelength is shown in Fig. S6 for a single, coated rod and different silver shell thicknesses. Two peaks can be observed in the spectrum. One at about 400nm, corresponding to the so-called transverse mode and the second in the range of 600nm - 700nm corresponding to the longitudinal mode. The transverse mode gets a red shift for thicker silver coatings and the intensity increases. The longitudinal is blue shifted with increasing silver layer thickness. This is in good agreement with the previous results.[(5)] Furthermore, it can be observed that the shift of the resonances is sensitive to the thickness of the silver layer.

The peak to dip normalised CD signal for the NR-NR arrangement in X- and L-shape is shown in Fig. S7. Here, mainly two peaks occur in the CD signal, the first in the range of 550 nm – 650 nm and the second one within 650 nm – 720 nm. For larger silver layer thicknesses, both resonances receive a blue shift, which, however, is less pronounced with increasing layer thickness. For large coating thicknesses, there is an additional peak at around 450nm. This might be caused by coupling of the transversal modes of the Au/AgNRs.

This journal is © The Royal Society of Chemistry 20xx

Please do not adjust margins

Figure S6. Numerical result of the normalized total extinction as function of the illumination wavelength for a single coated rod with homogeneous silver shell thickness t_{sh}, varied from 5 nm in 2 nm steps up to 23 nm.

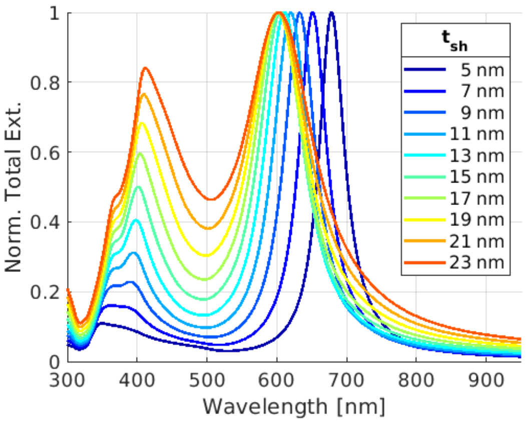

Figure S7. Numerically obtained normalised circular dichroism (CD) signal as function of illumination wavelength for different,

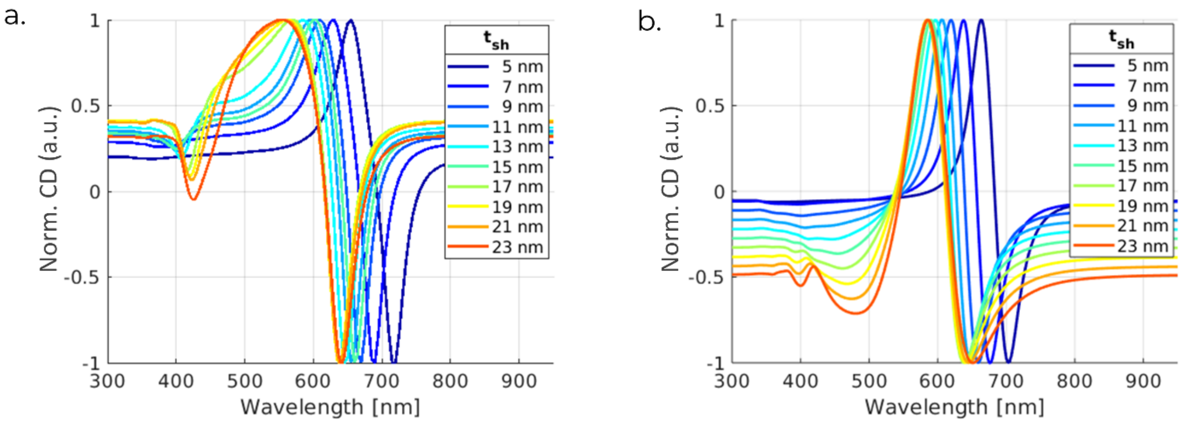

homogeneous silver shell thicknesses, t_{sh} varied from 5 nm up to 23 nm in 2 nm steps. a) NR-NR arrangement in X-shape, b) NR-NR arrangement in L-shape.

This journal is © The Royal Society of Chemistry 20xx

Please do not adjust margins

Please do not adjust margins

L-shape with imperfect angle

When assembling the L-shape dimers, it may happen that the angle between the two rods is not exactly 90° (Fig. S4). In the event that it is smaller, the L-shaped arrangement resembles the X-shape more and more. We simulated the L-shape arrangement for the 6mM variant of Au/AgNRs with different angles between the two rods (90°, 80°, 70° and 60°) to determining the influence of the angle on the CD spectra. The results are shown in Fig. S8. For smaller angles, the shape profile of the normalised CD begins to resemble the experimental results (Fig. 4b), with the dip around 400nm becoming more pronounced at lower angles.

Figure S8. Normalised CD in dependence of the wavelength for AuAg NR arrangement in L-shape for 6mM and different angles.

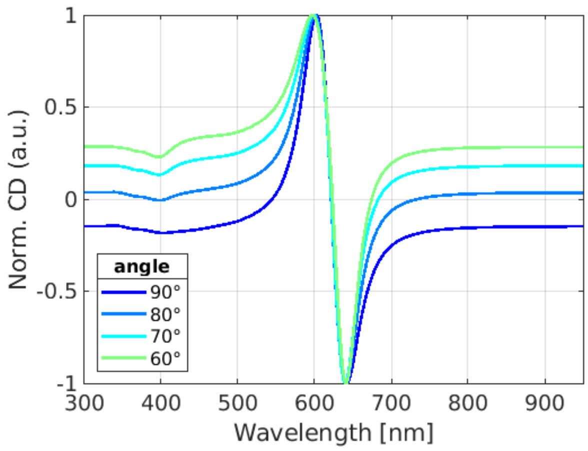

Please do not adjust margins

Please do not adjust margins

Supplementary note S3 – Shell thickness analysis

The nanoparticles' polydispersity, especially in the width, increases with the growth of thicker Ag shells (Fig. S8, right column). The synthesis of nanocrystals, in this case the Ag shell, is extremely sensitive to growth conditions. This inhomogeneity in the growth of nanocrystals has been widely studied, especially for gold nanocrystals.[(1,6)] Several ways have been suggested to improve the synthesis, including careful control over parameters like temperature during the growth phase [(6)], as well as addition of specific additives than can influence the monodispersity of the particles during growth.[(7)]

This journal is © The Royal Society of Chemistry 20xx

Please do not adjust margins

Size distributions of all NRs were determined by analyzing transmission electron microscopy (TEM) images. The dimension analysis of all images was done using ImageJ software (https://imagej.nih.gov/ij/).

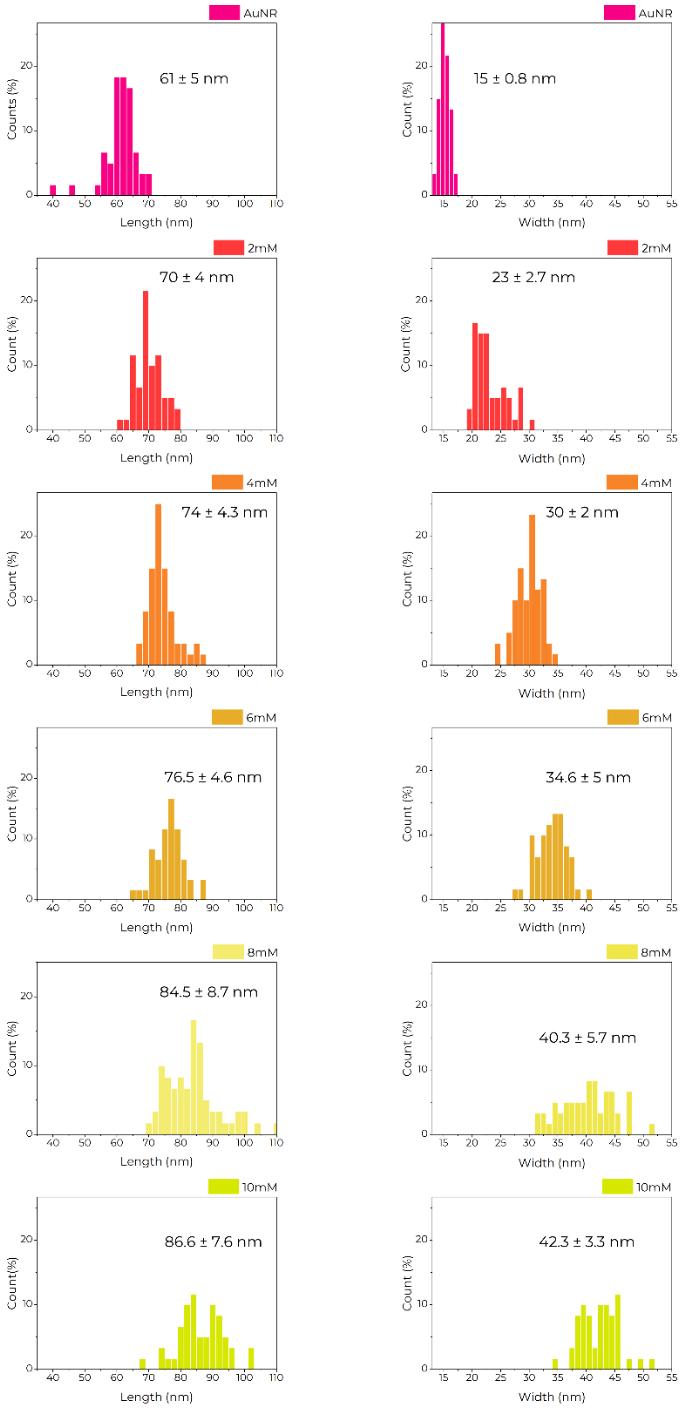

Figure S9. Size distribution of AuNRs and Au/AgNRs synthesized using different Ag^+ concentrations.

Supplementary note S4 – Spectral analysis of shifting LSPR

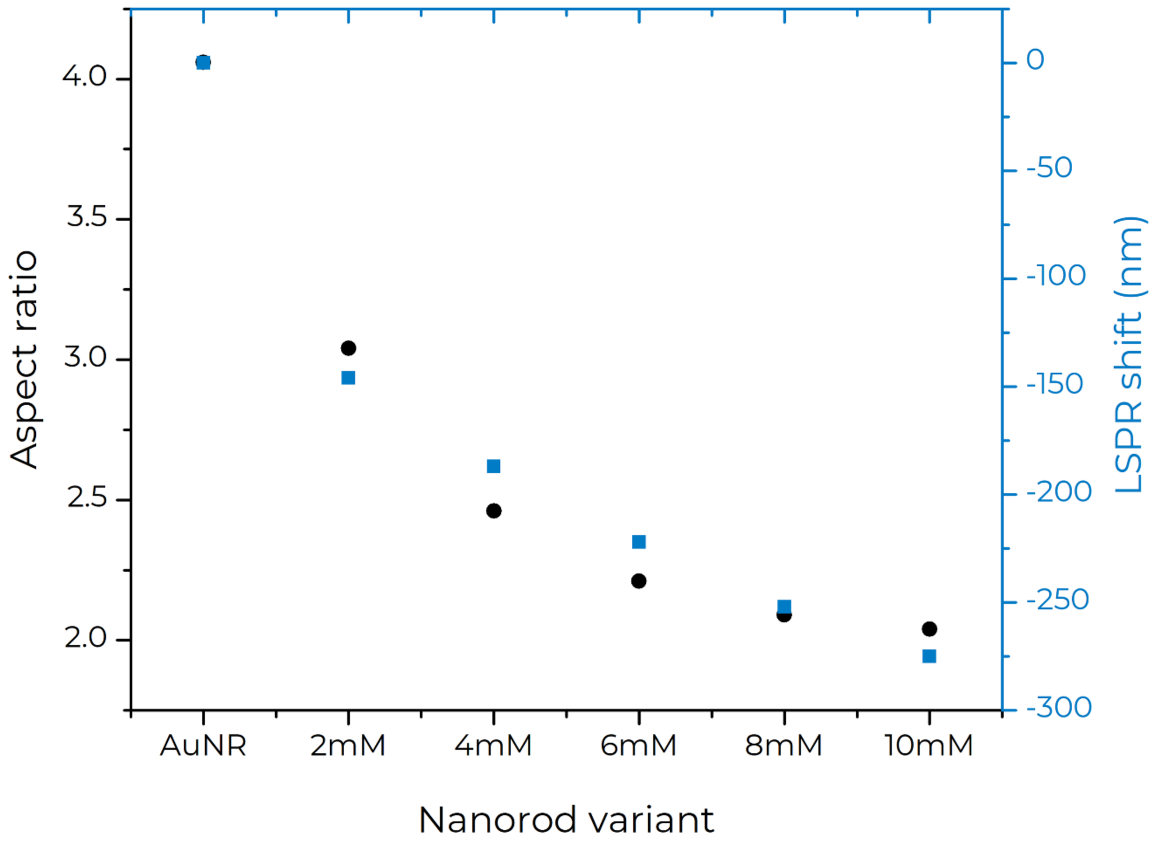

Figure S10. Variation in aspect ratio and LSPR peak of the Au/AgNRs relative to AuNRs.

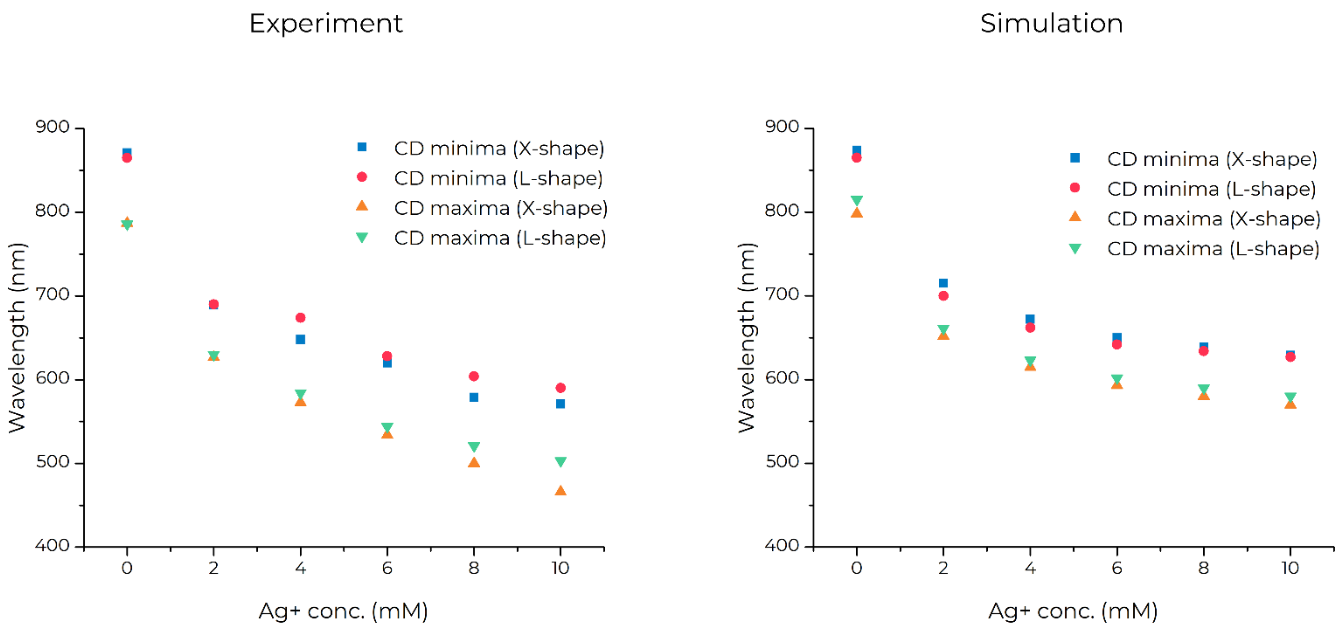

Figure S11. Comparison between theoretical and experimental CD spectra analysing the spatial tuning of the spectral maxima and minima.

 This journal is © The Royal Society of Chemistry 20xx

Please do not adjust margins

Supplementary note S5 – Thin shells

The realization of very thin Ag coatings, smaller than 5nm are technically not trivial. The experiments have shown that these arrangements do not remain sufficiently stable to be able to carry out measurements. Therefore, coating thicknesses from 1nm to 5nm are investigated numerically. For a single coated rod, the model and the numerical simulation procedure from Section S2-Numerical Section is used. The Ag coating is varied from 1nm to 5nm. The dimensions are given in Table S3. The results for the normalised total extinction is shown in Figure S11, where $t_{sh} = 0$ corresponds to a pure gold rod without silver coating. For thicker coatings the resonance in the higher wavelength regime is blue shifted and a second resonance around 350nm is occuring. The curves lie within the range between the pure Au NR and the smallest AuAg NR with smallest coating in Fig. 1.

For the X-shape and L-shape arrangements, the models and the numerical simulation procedure from Section S2-Numericlal Section are used. The Ag coating thickness is varied homogeneously from 1nm to 5nm, for dimensions see Table S3. The results of the normalised Circular Dicroism (CD) for X- and L-shape with different shell thickness are depicted in figure S 12. A layer thickness of $t_{sh} =$ 0 nm corresponds to pure gold rods. The CD has two peaks, the transverse mode in the range of 650nm - 850nm and the longitudinal mode in the range of 700nm - 900nm. For thicker Ag films, both peaks are blue shifted. The thicker the Ag layer, the more blue shifted is the CD. In addition, the distances between the CDs becomes smaller. The CD of the thinnest silver coating is very close to the result of the CD for pure Au rods (t_sh = 0nm). For both arrangements the curves lie within the range between the pure Au NR and the smallest AuAg NR with smallest coating in Fig. 4.

Table S3 Homogenous silver coating with $t_{sh} = t_d = t_h$ starting from 1 nm in 1 nm steps up to 5 nm, corresponding to the simulations of Fig. S11, Fig. S12 and the resulting diameter d_o and height h_o of the outer rod.

Case	1	2	3	4	5
$t_{sh} = t_d = t_h$ [nm]	1	2	3	4	5
d_o [nm]	17	19	21	23	25
h_o [nm]	63	65	67	69	71

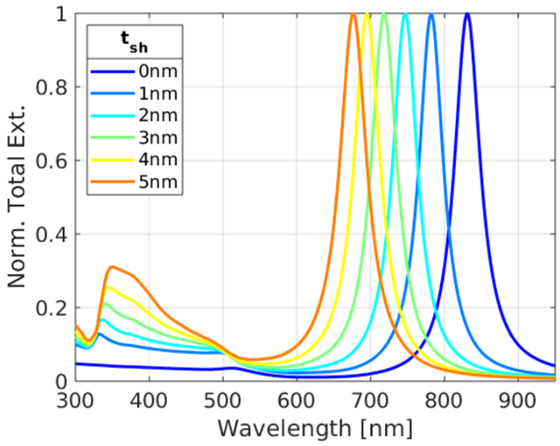

Figure S12. Numerically obtained total extinction as function of illumination wavelength for a single coated rod with different, homogeneous silver shell thicknesses, t_{sh} varied from 1 nm up to 5 nm in 1 nm steps and normalized total extinction for pure gold rods ($t_{sh} = 0$ nm). a) NR-NR arrangement in X-shape, b) NR-NR arrangement in L-shape.

This journal is © The Royal Society of Chemistry 20xx

Please do not adjust margins

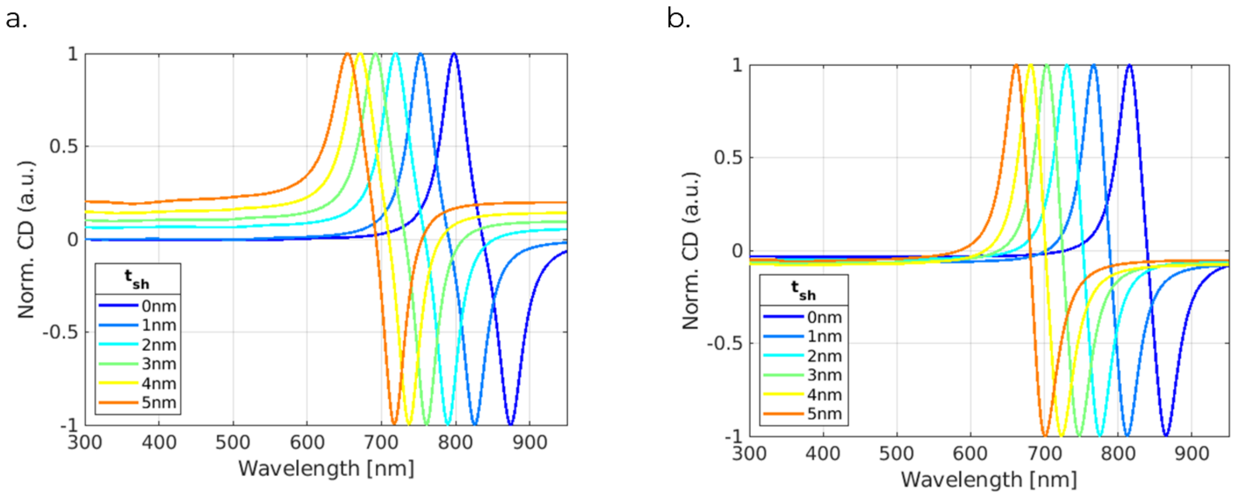

Figure S13. Numerically obtained normalised circular dichroism (CD) signal as a function of illumination wavelength for different, homogeneous silver shell thicknesses, t_{sh} varied from 1 nm up to 5 nm in 1 nm steps and normalised CD for pure gold rods ($t_{sh} = 0$ nm). a) NR-NR arrangement in X-shape, b) NR-NR arrangement in L-shape.

This journal is © The Royal Society of Chemistry 20xx
Please do not adjust margins

Please do not adjust margins

Supplementary note S5 – Thick shells

The evolution of the aspect ratio of the AuAgNRs with increasing Ag^+ concentration is tied to the aspect ratio of the underlying core AuNRs. For the AuNRs used in this paper (with dimensions ~ 61 nm x 15 nm), using 12mM Ag^+ in the growth solution led to Au/AgNRs that were almost isotropic (Fig. S11a,b), which makes it difficult to define the length vs width and thus, makes the calculation of the aspect ratio unreliable.

This isotropic nature of the Au/AgNRs is also mirrored in their extinction spectra, which no longer exhibits separate longitudinal and transverse modes but rather shows an almost total overlap between the two (Fig. S11c).

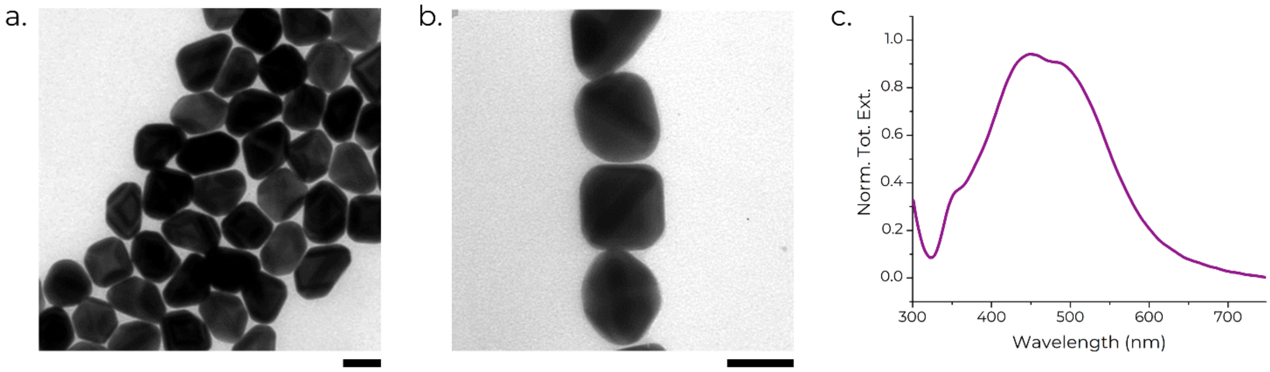

Figure S14. Growing thick Ag shells. a) TEM image of AuAgNRs grown using 12mM Ag^+. b) A close-up of the AuAgNRs. c) Normalized total extinction of the Au/AgNRs. Scale bars : 50 nm.

This journal is © The Royal Society of Chemistry 20xx

Please do not adjust margins

Please do not adjust margins

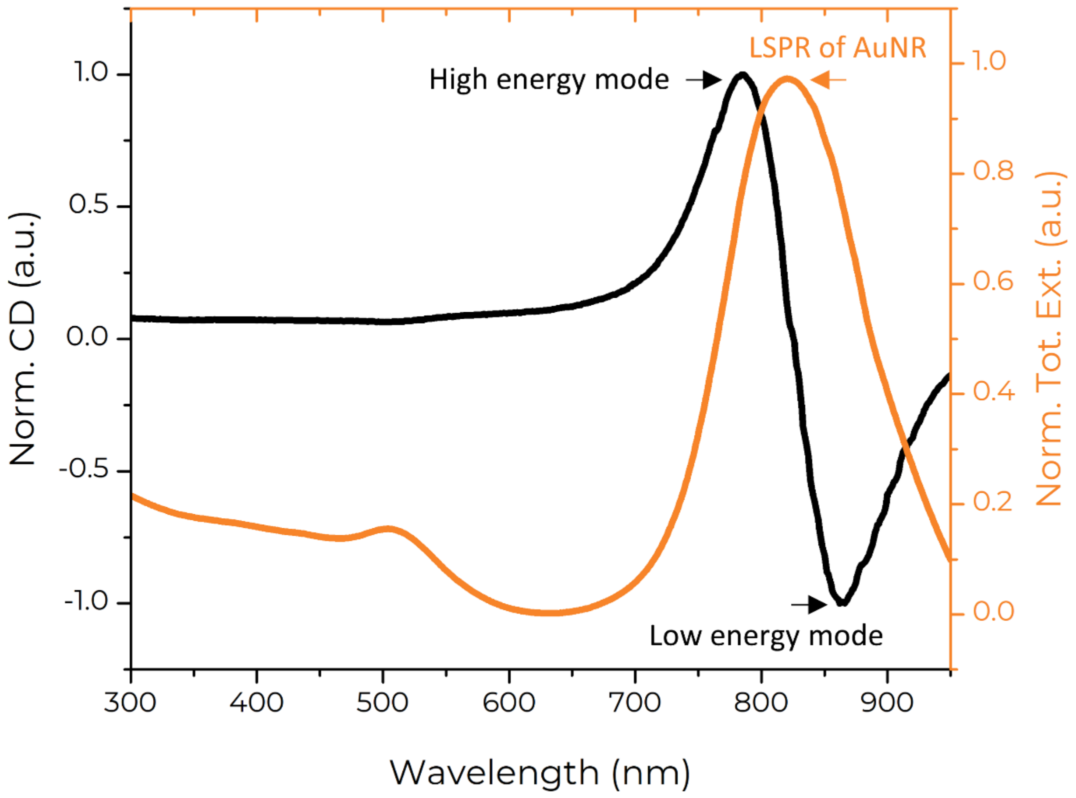

Figure S15. An overlay of the CD and normalized extinction spectra of L-shape dimer made with AuNRs, depicting their relative spectral positions. The LSPR hybridization modes in the chiral dimer are labelled for the CD spectra.

 This journal is © The Royal Society of Chemistry 20xx

Please do not adjust margins

Please do not adjust margins

DNA Origami design

The 2-layer sheet structure is designed to have an array of sites on both sides of the origami from which the 3' site of a staple can be extended. The green staples are designed such that their 3' ends face the 'bottom' face. Similarly, for the red staples the 3' ends face the 'top' face. The staples on the edges are extended with three C's to prevent base stacking between origami structures. Eight A's are extended as anchors to bind to functionalised nanorods.

In the accompanying list of oligos, the 'core' staples are common to both the X- and the L-shape designs. The 'staples' are oligos without any extended part,

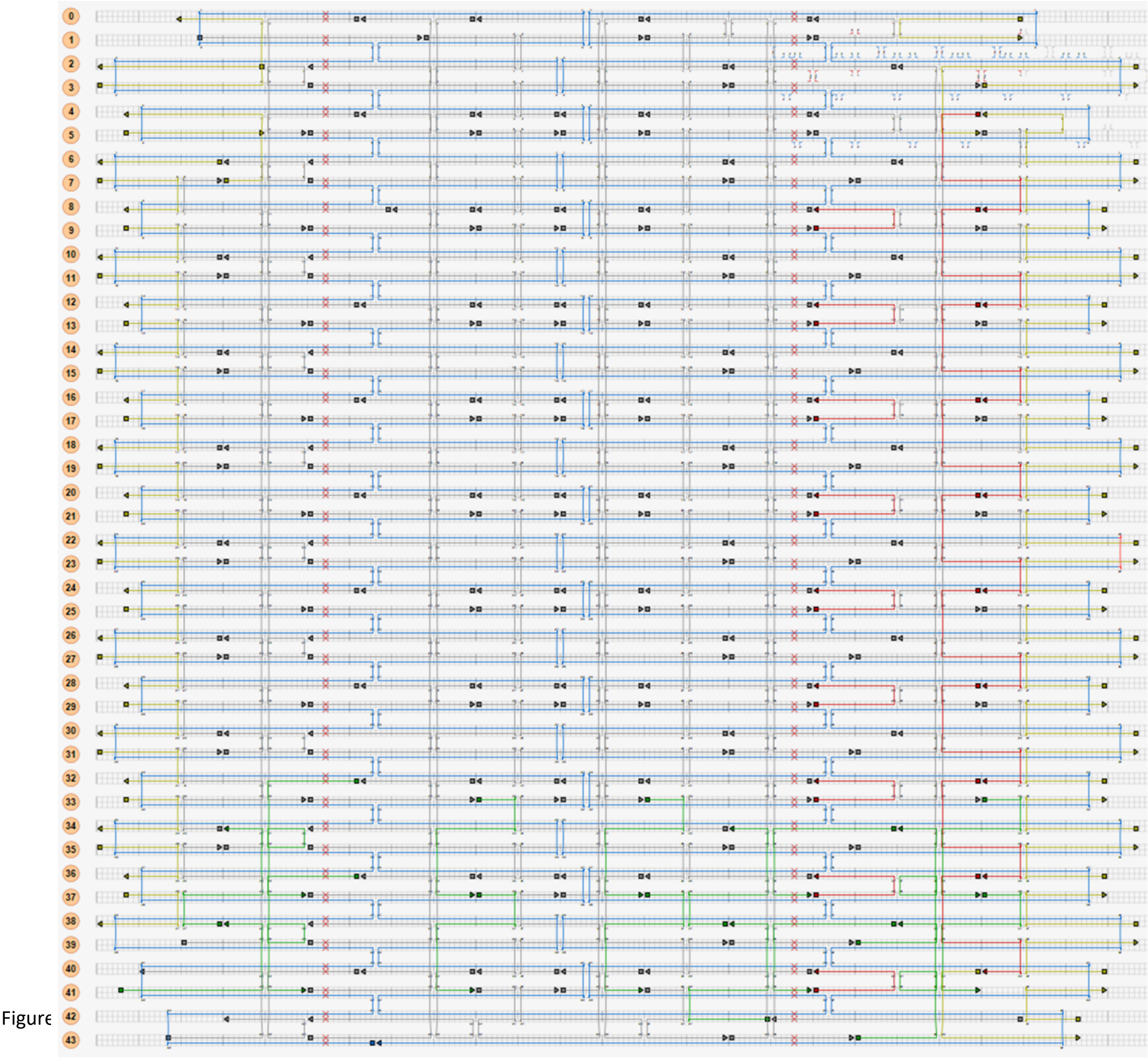

Figure

while the 'handles' are oligos with a polyA extension.

Please do not adjust margins

Please do not adjust margins

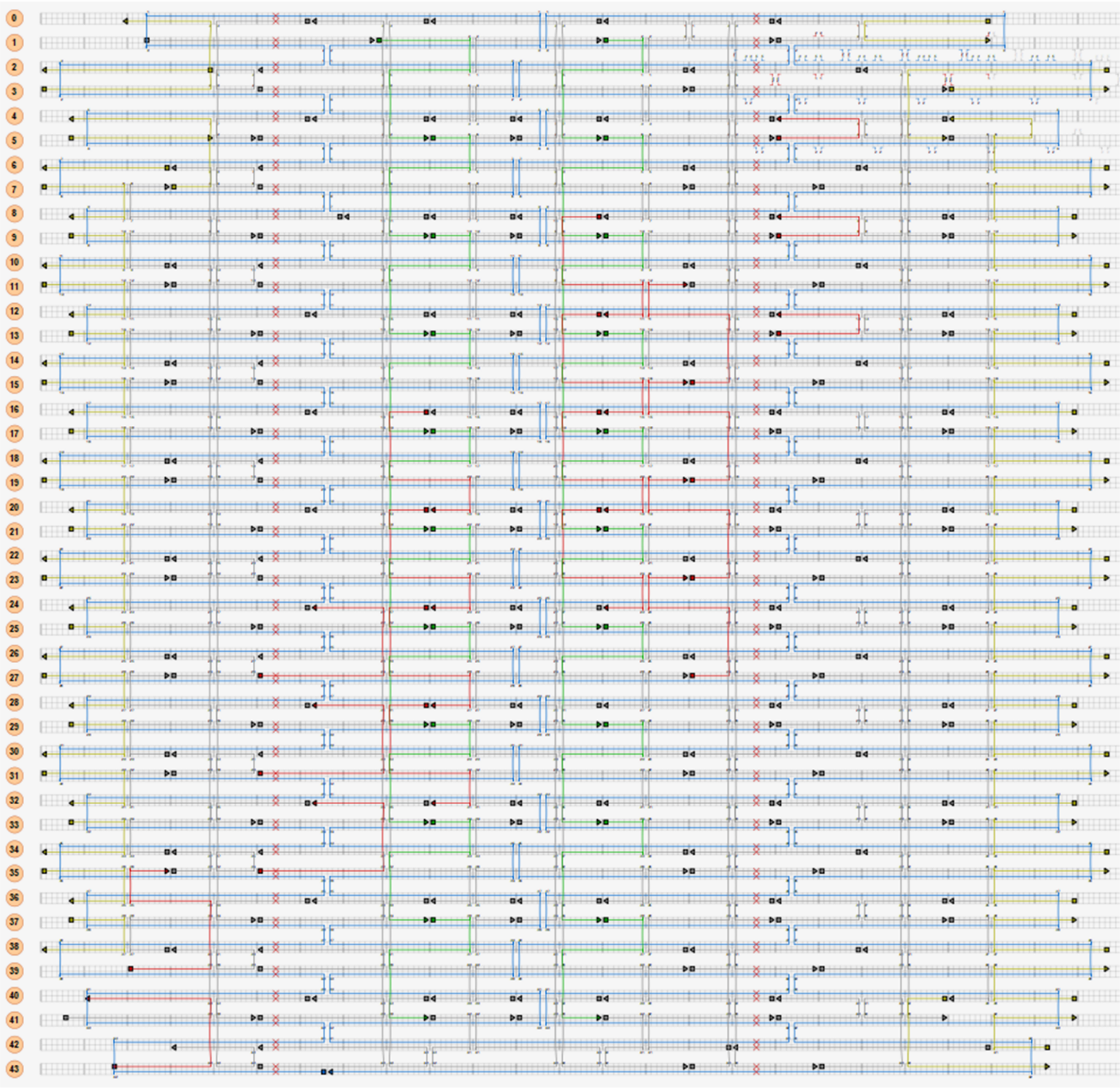

Figure S17. Cadnano design for the 2-layer sheet structure with anchors to form the X-shape metamolecule.

References

1. González-Rubio G, Kumar V, Llombart P, Díaz-Núñez P, Bladt E, Altantzis T, et al. Disconnecting Symmetry Breaking from Seeded Growth for the Reproducible Synthesis of High Quality Gold Nanorods. ACS Nano. 2019 Apr 23;13(4):4424–35.

2. Gür FN, Schwarz FW, Ye J, Diez S, Schmidt TL. Toward Self-Assembled Plasmonic Devices: High-Yield Arrangement of Gold Nanoparticles on DNA Origami Templates. ACS Nano. 2016 May;10(5):5374–5382.

3. Lan X, Lu X, Shen C, Ke Y, Ni W, Wang Q. Au Nanorod Helical Superstructures with Designed Chirality. J Am Chem Soc. 2015 Jan 14;137(1):457–62.

4. Pomplun J, Burger S, Zschiedrich L, Schmidt F. Adaptive finite element method for simulation of optical nano structures. physica status solidi (b). 2007;244(10):3419–34.

 This journal is © The Royal Society of Chemistry 20xx

Please do not adjust margins

Please do not adjust margins

5. Nguyen L, Dass M, Ober MF, Besteiro LV, Wang ZM, Nickel B, et al. Chiral Assembly of Gold–Silver Core–Shell Plasmonic Nanorods on DNA Origami with Strong Optical Activity. ACS Nano. 2020 Jun;14(6):7454–7461.

6. Scarabelli L, Sánchez-Iglesias A, Pérez-Juste J, Liz-Marzán LM. A “Tips and Tricks” Practical Guide to the Synthesis of Gold Nanorods. J Phys Chem Lett. 2015 Nov 5;6(21):4270–9.

7. Ye X, Jin L, Caglayan H, Chen J, Xing G, Zheng C, et al. Improved Size-Tunable Synthesis of Monodisperse Gold Nanorods through the Use of Aromatic Additives. ACS Nano. 2012 Mar 27;6(3):2804–17.

This journal is © The Royal Society of Chemistry 20xx

Please do not adjust margins
